# Genetic and Environmental Factors Jointly Impact Leaf Phenolic Profiles of *Iris variegata* L.

**DOI:** 10.3390/plants10081599

**Published:** 2021-08-04

**Authors:** Uroš Živković, Stevan Avramov, Danijela Miljković, Nataša Barišić Klisarić, Ljiljana Tubić, Danijela Mišić, Branislav Šiler, Aleksej Tarasjev

**Affiliations:** 1 Department of Evolutionary Biology, Institute for Biological Research “Siniša Stanković”, National Institute of Republic of Serbia, University of Belgrade, Bulevar Despota Stefana 142, 11060 Belgrade, Serbia; stevan@ibiss.bg.ac.rs (S.A.); danijela.miljkovic@ibiss.bg.ac.rs (D.M.); natasa@ibiss.bg.ac.rs (N.B.K.); tarasjev@ibiss.bg.ac.rs (A.T.); 2 Department of Plant Physiology, Institute for Biological Research “Siniša Stanković”, National Institute of Republic of Serbia, University of Belgrade, Bulevar Despota Stefana 142, 11060 Belgrade, Serbia; tubic@ibiss.bg.ac.rs (L.T.); dmisic@ibiss.bg.ac.rs (D.M.); branislav.siler@ibiss.bg.ac.rs (B.Š.)

**Keywords:** secondary metabolites, genetic variability, light treatments, seasonal variability, Hungarian iris

## Abstract

A plant’s main mechanism to diminish the effects caused by high free radical levels generated during high irradiance is the synthesis of various secondary metabolites. In addition to interspecies differences, their concentrations may be influenced by genetic, ontogenic, morphogenetic or environmental factors. We investigated the influence of genetic (genotypes from different natural habitats) and environmental (contrasting light regimes as well as successive parts of the vegetation period) variability on the accumulation of 10 selected phenolic compounds (phenolic acids, flavonoids, and xanthones) in *Iris variegata* genotypes. Genotypes originated from either sun-exposed or shaded natural habitats were transplanted to two experimental light treatments (high light intensity with a higher R/FR ratio and low light intensity with a lower R/FR ratio). Significant impacts of both genetic and environmental seasonal variability (spring, summer and fall during the vegetation period) on phenolic compound profiles were detected. Their highest amounts were detected in spring. The magnitude of difference between light treatments (high vs. low light intensity) and the direction of this change varied depending on the secondary compound class. Phenotypic correlations among the 10 analyzed secondary metabolites differed across the experimental light treatments and their number decreased from spring to fall.

## 1. Introduction

Light is one of the most dynamic components of a plant’s environment, which differs strongly both spatially and temporally. As plants are sessile organisms, they need to cope with such variations and acclimate through their morphology, anatomy, physiology and metabolism, as well as in flowering phenology and reproductive output [1,2,3,4].

High fluxes of solar radiation often lead to the overproduction of reactive oxygen species (ROS), causing function loss of both proteins and lipids, and DNA damage as well [5]. Higher plants developed protection mechanisms to diminish the effects caused by increased amounts of free radicals (superoxide, hydrogen peroxide, the hydroxyl radical, and singlet oxygen) generated during high solar irradiance [6]. A detoxification mechanism engaged is the synthesis of secondary metabolites, differing substantially in terms of biosynthetic origin and chemical structure [7,8]. Metabolic flux, the total content and relative proportions of secondary metabolites present in a plant may be influenced by genetic, ontogenic, morphogenetic and environmental factors [9,10]. They also show seasonal and daily variations that can be visible on the intraplant, interplant, and interspecies levels [11]. Therefore, induction of important biosynthetic regulatory enzymes for these biologically active compounds can be further regulated by red/far-red (R/FR) light ratio, circadian rhythm, different developmental stages, phenology, high/low temperature, altitude, water and nutrient availability, UV radiation, pollution, mechanical stimuli and attacks by herbivores or pathogens [12,13,14,15]. Differences in the results obtained on a wide range of species studying various abiotic and biotic factors highlight the need for their full dose-response curves to provide a more complete understanding of plant chemical responses to environment signals [4,16,17]. Congruent studies should be performed to perceive the patterns underlying the variation in quantities and distribution of the vast number of secondary metabolites.

Genus *Iris* contains around 300 species and metabolic profiling was performed for only 10 of them [18]. The most abundant secondary compounds within the genus are: isoflavonoids, flavones, triterpenes, iridals, xanthones, quinones, peltogynoids and stilbenes [19,20,21,22]. A relative amount of these chemicals can vary within a species and between species. Phytochemical profiling in different plant parts was performed in three *Iris* species from Serbia: *I. humilis* L., *I. pumila* L. and *I. variegata* L. [23]. A low-resolution analysis presented in this study showed that phenolic compounds were represented by four structurally distinct groups: xanthones, flavonoid C-glucosides, flavonoid O-glucosides, and isoflavones and their derivatives. Studies that explicitly considered diversity in secondary metabolite chemistry in the intraspecific level as well as in different environmental contexts are quite rare and were not conducted for these species.

It was reported that genotypes of several *Iris* species displayed a remarkable variability in response to different light regimes [1,3,24,25]. Besides previous insights into the existing variability at different levels of biological organization (molecular, physiological, anatomical, morphological and population), assessment of the presence of individual secondary metabolites and their quantitative variation are of a great importance and interest as well. To the best of our knowledge, literature data on the effects of the quality and quantity of light on the composition of biologically active compounds in these plants are not available. *I. humilis* and *I. pumila* are strictly protected species in the Republic of Serbia, therefore multifactorial experiments that require a large number of genotypes are possible only after obtaining a special permit from Ministry of Environmental Protection. Therefore, we conducted this study only on existing samples of *I. variegata* natural genotypes already acclimated and grown in an experimental setting.

The study is based on establishing a multifactorial common garden experiment using *I. variegata* (Hungarian, variegated or multicolored iris) towards analyzing genetic (genotypes originating at two natural habitats) and environmental (different experimental light regimes and different parts of the vegetative period) sources of chemodiversity as well as correlations between metabolite amounts that were not analyzed before.

The following questions were addressed: does the content of the studied phenolic compounds differ between two experimental light treatments? Is there an effect of different genotypes and habitats on the variation of the target compounds? How did the amounts of monitored compounds change during vegetation period? Does the pattern and strength of phenotypic correlations between secondary metabolites vary between two light treatments and through the vegetative period?

The experiment on *I. variegata* could provide guidelines for future research on protected *Iris* species with the aim of their better protection and a much greater understanding of climate change-related variations in the relationships between secondary metabolism and resistance to abiotic stress.

## 2. Results

### 2.1. Phenolic Acids

**Figure 1:**
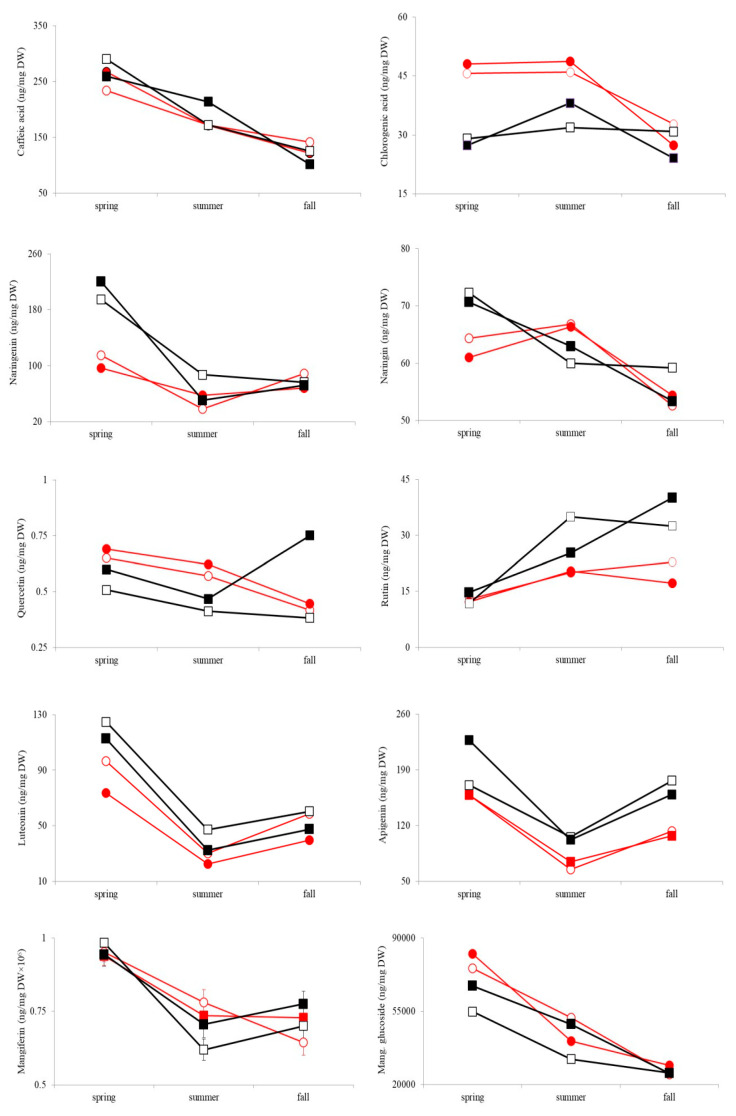
Mean leaf content of caffeic acid, chlorogenic acid, naringenin, naringin, quercetin, rutin, luteolin, apigenin, mangiferin and mangiferin glucoside in *Iris variegata* genotypes originating from sun-exposed habitat (□—in low light treatment; ○—in high light treatment) and shaded habitat (■—in low light treatment, ●—in high ligh treatment) in tree parts of vegetative period (spring, summer, and fall).

Plants from both high and low light treatments contained around 5–9-fold more caffeic than chlorogenic acid in all consecutive seasons (Figure 1). The difference between two treatments was significant for both phenolic acids (Table 1). Caffeic acid amounts were higher in the leaves of the plants growing in low compared to those grown in high light treatment (Figure 1). Significant treatment × genotype interaction indicates the genetic variability of phenotypic plasticity to light conditions for the observed trait (Table 1). Results also showed that the differences between the seasons were significant and treatment-specific (season × treatment) (Table 1, Figure 1). Caffeic acid amounts during spring and summer were higher in the extracts obtained from the genotypes growing in low light treatment, compared to high light treatment (Figure 1). The mean values of the observed trait changed in different ways in genotypes across seasons (season × genotype) and across seasons and treatments (season × treatment × genotype) (Table 1). Profile analyses revealed a significant mean effect in all three time intervals, which clearly showed that there were significant differences in the mean caffeic acid content among the seasons (Table 1, Figure 1).

Significantly higher amounts (35–65%) of chlorogenic acid were obtained in leaves from the high light treatment, compared to leaves from the low light treatment (i.e., phenotypic plasticity) (Table 1, Figure 1). Differences between seasons were significant and treatment-specific (season × treatment) (Table 1). Chlorogenic acid content during spring and summer was higher in the extracts obtained from the genotypes growing in high light treatment, in comparison to low light treatment (Figure 1). Analyses revealed significant differences in the amounts of chlorogenic acid in the leaves between spring and summer, and between spring and fall (Table 1).

### 2.2. Flavonoids 

The difference between the two treatments was significant and the amount of naringenin was higher in the extracts obtained from the genotypes growing in low light treatment comparing to high light treatment (Table 1, Figure 1). The effect of genotype (H) was also significant, indicating genetic variability within habitats for naringenin content (Table 1). The significant season effect indicates variations of naringenin concentrations between spring, summer and fall. Significant season × treatment interaction indicates the seasonal variability for phenotypic plasticity of naringenin content (Table 1, Figure 1). During spring, the plants accumulated significantly higher concentrations (95%) of naringenin in low light treatment, compared to high light treatment (Table 1, Figure 1). Significant season × genotype (H) interaction revealed that differences in naringenin concentrations among genotypes depended on the vegetation season (Table 1). Profile analyses indicated significant differences in the mean naringenin concentration among all three seasons (Table 1, Figure 1). The amount of this flavonoid was the highest during spring in both light treatments (Figure 1).

The naringin concentration was not significantly influenced by the experimental light treatments or a season (Table 1). However, we must take into consideration that during the fall this flavonon was detectable in only a few leaf samples, which produced an incoherent picture with only one interaction (season × treatment × habitat), showing a significant impact (Table 1). This double interaction implied that the variability of plasticity between the seasons for the observed trait depended on the type of habitat of *I. variegata* (Figure 1).

In contrast to some previously mentioned metabolites in which different light treatments affected their content, quercetin amounts did not differ significantly across the experimental light treatments (Table 1). There was a significant effect of the genotype (H) which indicates that there was significant genetic variability within habitats for this trait. Analysis also showed a significant season effect and variability of plasticity between the seasons for this trait (Season × Treatment) (Table 1). The quercetin amount was decreasing throughout the vegetative seasons in all samples of *I. variegata*, except for the genotypes originated from the shaded habitat and growing in low light treatment (Figure 1). Individual ANOVAs computed on each of the three contrast variables showed that quercetin amounts were significantly different between spring and summer, and summer and fall (Table 1, Figure 1).

Light treatment did not have a significant effect on the foliar rutin content of *I. variegata* (Table 1). The significant effect of the genotype (H) on this trait was observed, implying significant genetic variability for rutin concentration in *I. variegata* leaves within habitats (Table 1). A significant season effect and increase in rutin content in leaves of *I. variegata* was observed from spring to fall (94–118%) (Figure 1). Significant differences were observed between spring and summer as well as between summer and fall (Season effect) (Table 1). The responses of *I. variegata* plants to the experimental treatments were significantly influenced by seasons (season × treatment interaction) with higher rutin amounts accumulated in low light treatment than in high light treatment during summer and fall (Table 1, Figure 1). 

A significant effect of treatment (i.e., phenotypic plasticity) was recorded for luteolin (Table 1). The amounts of the studied compound were higher in the extracts obtained from the low light treatment, compared to the high light treatment (Table 1, Figure 1). *I. variegata* genotypes originating from the sun-exposed habitat exhibited a higher concentration (27%) of luteolin compared to the plants from shaded habitat regardless of the experimental light treatment (significant habitat effect) (Table 1, Figure 1). ANOVA for this metabolite also revealed statistically significant differences between genotypes of *I. variegata* (significant genetic variability within habitats) (Table 1). The results of ANOVA on the differences of consecutive seasons revealed a statistically significant season effect, indicating that the mean luteolin content significantly differed among spring, summer and fall (Table 1, Figure 1). The highest concentration of luteolin was recorded in spring, decreased during summer and increased in fall (Figure 1). 

Apigenin is another flavone compound identified and quantified in *I. variegata* leaves. Treatment effect was not significant, but the effect of the genotype (H) showed significant genetic variability within the habitats (Table 1). Results showed a significant season effect and variability of plasticity between the seasons for this compound (season × treatment) (Table 1). Profile analysis revealed significant differences in the mean apigenin content between all three seasons (Table 1, Figure 1). Changes in apigenin amounts during seasons followed the trend of changes observed for luteolin with the highest concentration recorded in spring, a significant decrease in summer, and another increase in fall (Figure 1). The season × genotype interaction indicated that genotypes changed their ranks in different seasons, concerning apigenin content (Table 1). 

### 2.3. Xanthones

Accumulation of mangiferin did not differ significantly between the contrasting light treatments (Table 1). However, the analysis showed a significant season effect. Accumulation of this metabolite in leaves of *I. variegata* in response to high and low light treatment differed across the three seasons (significant season × treatment interaction) with the summer content being higher in the extracts obtained from the genotypes growing in the high light treatment, compared to the low light treatment (Table 1, Figure 1). Profile analysis showed significantly higher (32–37%) mangiferin amounts in spring compared to both summer and fall (Table 1, Figure 1).

Mangiferin glucoside was the most abundant phenolic compound quantified in *I. variegata* samples analyzed within the present study with amounts reaching approximately 80 μg/per 100 mg DW, which is almost 300-fold higher quantity than the second most abundant compound recorded (caffeic acid). The difference between the two light treatments was significant and the amounts of the observed compound were 20% higher in the extracts obtained from the genotypes growing in high light treatment compared to low light treatment (Table 1, Figure 1). *I. variegata* leaves also showed a significant variability between genotypes within the habitat (significant genotype (H) effect) (Table 1). Results indicated the existence of significant differences in mangiferin glucoside in different vegetation seasons (Season effect, Table 1). The concentration of mangiferin glucoside significantly decreased up to 60% during the three consecutive vegetative seasons (from spring to fall) (Figure 1). The results also showed a significant interaction between season and treatment that indicates the variability of the plasticity of the trait compound accumulation during the observed seasons (Table 1) as well as the dependence of seasonal variation on differences between plant genotypes (interaction season × genotype (H)) (Table 1). Significant second-order interaction, season × treatment × habitat, confirms that the variability of plasticity between the seasons for the observed trait depends on the type of habitat in which the *I. variegata* plants originate (Table 1).

### 2.4. Correlations

**Figure 2:**
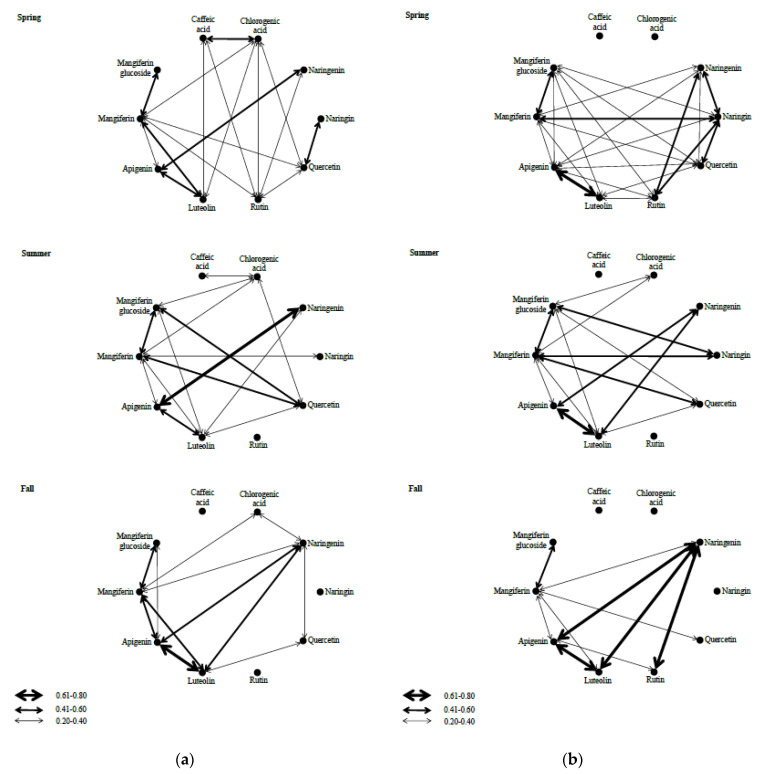
Statistically significant correlations between 10 targeted metabolites quantified in *I. variegata* plants grown in high (**a**) and low (**b**) light treatments across three seasons of the vegetation period (spring, summer, and fall).

Under high light treatment, positive significant correlations were revealed between the amounts of chlorogenic and caffeic acid during spring and summer (Figure 2, Tables S1 and S2). Under low light treatment a significant correlation between phenolic acids and flavonoids was not recorded (Figure 2, Tables S1–S3). On the contrary, in the high light treatment, during spring, a significant correlation was obtained between caffeic acid and rutin as well as between caffeic acid and luteolin (Figure 2, Table S1). A significant correlation was also observed in high light treatment in the summer season between chlorogenic acid and both xanthones (Figure 2, Table S2). The correlations between xanthones were significant and positive in both treatments and seasons (Figure 2, Tables S1–S3). The quantitative content of mangiferin is highly correlated with quercetin, luteolin, and apigenin in both treatments as well as in all three seasons of the vegetative period (Figure 2, Tables S1–S3). In low light treatment a significant correlation was detected between mangiferin glucoside and flavonoids (naringin, quercetin, and luteolin) during spring and summer (Figure 2, Tables S1 and S2). In high light treatment a significant correlation was detected between mangiferin glucoside and two flavonoids, quercetin and luteolin, only in summer (Figure 2, Table S2). A significant correlation between luteolin and apigenin was observed in both treatments across the three seasons (Figure 2, Tables S1–S3). The number of statistically significant correlations markedly decreased through the vegetative period (Figure 2, Tables S1–S3).

## 3. Discussion

Facing frequent fluctuations of their primary energy source, plants developed adaptation mechanisms for the photosynthetic efficiency upon balance between different wavelengths [26]. Acclimations to environmental perturbations in many ways define the pivotal roles of secondary metabolites in processes important for maintaining structural rigidity and the fine regulation of homeostasis [6]. Secondary metabolites act as a chemical interface between plants and their environment, conditioning antioxidant activities through altered polarity, volatility, biological activity, and chemical stability in cells [27,28].

By comprehensively analyzing the effects of environmental light conditions and genetic factors while taking into account the origin and adaptive traits of *I. variegata* genotypes in their natural habitats, the present study explains alterations in the polyphenolics content during the adaptation of plants to changing environments, and brings novel insights into the intertwining role of secondary metabolism between genotype and phenotype. The targeted metabolomics approach adopting the UHPLC/(−)HESI-MS2 analysis enabled us to quantify several robust phytochemical classifiers from the group of polyphenolics and distinguish the effects of contrasting light treatments on studied genotypes. Although the selection of compounds for this study was based on the literature survey and the availability of analytical standards, their content, patterns of quantitative changes during vegetative seasons, and the ratio between individual secondary metabolites highlighted targeted compounds as reliable indicators of the secondary metabolism status in *I. variegata* leaves under different light conditions.

### 3.1. Variation in Phenolic Acids Content

The initial products formed during abiotic stress events triggered by transcriptional activation of the phenylpropanoid pathway are caffeic and chlorogenic acids [29]. It was shown that a wide range of environmental factors such as UV light, mineral deficiency and microbial pathogens increased chlorogenic acid levels in various plant tissues [30]. In the present study, plants from the high light treatment contained significantly higher levels of chlorogenic acid during the three seasons of the vegetative period. The accumulation of this free radical scavenger under high solar irradiance might represent an antioxidant response to stress caused by excess light [31,32]. The observed seasonal differences in the amounts of chlorogenic acid could arise from the response to increased free radical production.

A higher caffeic acid accumulation in low light treatment can be interpreted in terms of tissue-specific accumulation and perform a role in lignin biosynthesis. It is hypothesized that caffeic acid enhances lignin biosynthesis and therefore should be present in lower concentrations in highly irradiated cells [33]. Furthermore, caffeic acid is a building block for other more complex polyphenolics, including chlorogenic acid, and its amount in tissues is a result of its biosynthesis and its accumulation its further metabolization.

### 3.2. Variation in Flavonoids Content

A common denominator for all flavonoids is a flavanone naringenin, which can be converted into dihydroflavonols, leading to flavonols, leucoanthocyanidins, anthocyanidins, flavan-3-ols, proanthocyanidins [34]. In another branch, naringenin can be converted into flavones [35]. Significantly higher amounts of naringenin obtained in the treatment with lower light intensity and lower red/far-red ratio in *I. variegata*, are in agreement with theoretical expectations [36]. With a few exceptions, high naringenin content in *I. variegata* leaves under these conditions, was followed by low content of flavanone naringenin and flavonol quercetin, which are direct products of naringenin metabolization. These results indicate more intensive conversion of naringenin into naringin and quercetin under high light intensity, and thus more intensive flux through flavanone and flavonol branches of the flavonoid biosynthetic pathway.

Variability in flavonoid profiles may originate in auxin gradients that consequently affect the expression of phenotypes with strikingly different morphological and anatomical features [37]. Phenotypes exposed to direct sunlight irradiance (with few, small, and thick leaves) are rich in light-responsive flavonoids such as quercetin and luteolin. On the contrary, shaded plants (with long internodes, and large photosynthetically-active surface area coupled with reduced leaf thickness) are abundant with apigenin and negligible amounts of quercetin [38]. Regardless of the theoretical expectations, luteolin content in *I. variegata* leaves was significantly higher in low light treatment. On the other hand, mean apigenin quantities, determined in our study, changed in accordance with the ecophysiological predictions. Only one secondary metabolite, luteolin, showed a significant difference in its content between natural habitats; luteolin quantities were significantly higher in genotypes originating from sun-exposed habitats throughout the seasons and in both treatments (Figure 1, Table 1).

The majority of the analyzed compounds in both high and low light treatment accumulated depending on the season. Across the seasons, apigenin amounts changed to a greater extent than the concentration of rutin and quercetin, even though flavones are reported to have a lower antioxidant capacity than flavonols [39,40]. The synthesis of flavones in *I. variegata* was favored in low light treatment, contrary to the literature data [41,42,43]. Considering that flavonols show a greater antioxidant capacity, they should be more abundant under high light treatment. Both quercetin and rutin, for instance, protected wheat chloroplasts from thylakoid lipid peroxidation and pigment photo-oxidation under high illumination [44]. On the contrary, in our study, only quercetin displayed higher amounts under high light treatment.

The perception and signal transduction of red/far-red ratio regulates plant growth and development (shade-avoidance syndrome) [45]. In our previous study, leaves of *I. variegata* plants acclimated to either shade or full solar irradiance differed markedly in their morphological and ecophysiological characters [46]. Phytochrome-mediated shade avoidance responses also involve substantial transcriptome changes responsible for the synthesis of secondary metabolites [47], which could be the cause of an increased accumulation of the main flavonoid precursor naringenin in the low light treatment in our study.

### 3.3. Variation in Xanthones Content

Mangiferin and isomangiferin are, more or less, universally present in the *Iris* species [48,49]. Mangiferin is a heat-stable molecule and a natural pharmacologically active phytochemical with various bioactivities [50]. Besides the fact that mangiferin and mangiferin glucoside sporadically occur in other sections of the genus *Iris* and also in other genera belonging to the Iridaceae family, our results confirm that *I. variegata* is a rich source of these compounds. Similar to *Cyclopia genistoides* [51], the highest amounts of mangiferin in *I. variegata* leaves were recorded in spring, significantly decreased in summer, and again increased in fall. No significant effects of light intensity on the content of mangiferin was recorded throughout the seasons. However, in spring the content of mangiferin glycoside was significantly higher in leaves exposed to high light conditions.

### 3.4. Secondary Metabolites Correlation Patterns

**Figure 3:**
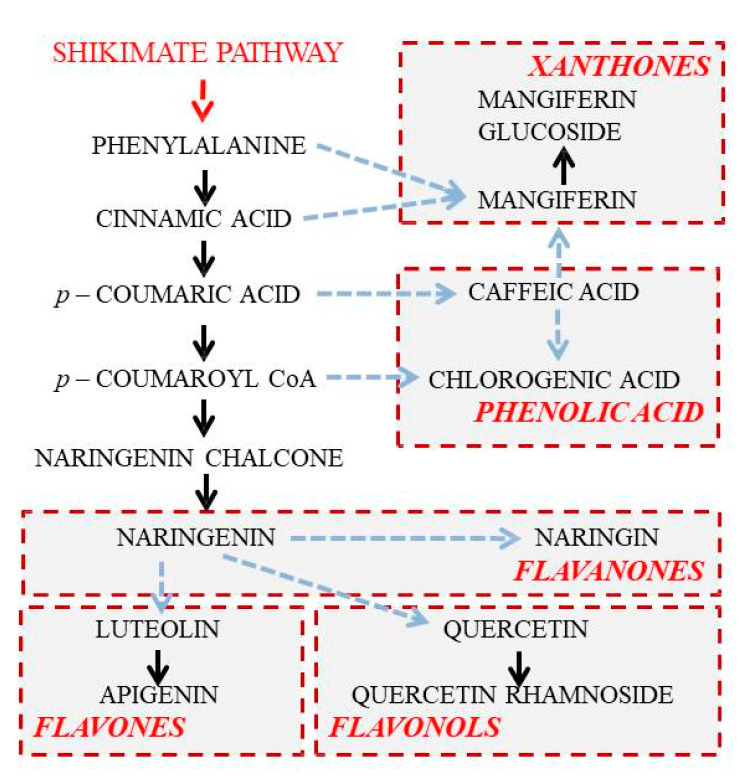
Proposed biosynthetic routes leading to phenolic compounds found in *I. variegata* leaves within the present study.

Correlations among traits can be a consequence of genetic, developmental, functional, physiological or ecological causes [52,53]. Taking into account that all the analyzed metabolites share, at least partially, a common biosynthetic route (Figure 3), it can be expected that they may manifest interconnection in expression. Flavanone, flavone and flavonol biosynthetic routes are branches of the flavonoid pathway, and they are most likely coordinately regulated in different tissues and organs and in response to various developmental and environmental cues, which can trigger changes in a number of biosynthetic pathways, their branches or individual steps. All these changes might lead to the modifications of the qualitative and quantitative composition of metabolites in tissues and organs.

Correlation patterns of the content of secondary metabolites changed in different light treatments as well as in different seasons of the vegetation period. Many of the significant phenotypic correlations among the ten target secondary metabolites decreased from spring to fall in both experimental light treatments.

The significant correlations between secondary metabolites that represent precursors and their products after a single enzymatic reaction (e.g., mangiferin/mangiferin glucoside, naringenin/apigenin, and apigenin/luteolin) were present in both light treatments and in all seasons of the vegetative period. On the other hand, significant correlations between some of the target metabolites that share a common biosynthetic path (e.g., caffeic/chlorogenic acid, naringenin/quercetin) were present in only one of the two light treatments and during only several seasons of the vegetative period. In order to understand correlation patterns in *I. variegata,* additional explanation that is not restricted to the biosynthetic pathways is needed.

All significant correlations between the analyzed secondary metabolites were positive in sign, even between compounds competing for the common precursors (e.g., naringin/quercetin, luteolin/quercetin), although we can expect a trade-off in their accumulation.

Changes in the number and intensity of significant correlations in different light treatments and parts of the vegetation period can have important consequences on the intensity and direction of the indirect selection processes in *I. variegata*. Future studies of the effect of environmental conditions on the expression of trait covariation should therefore be conducted in more than one environment. Due to these great differences in the correlation patterns in our experiment, and also the lack of similar data, it would be of general interest to investigate the expression patterns of both biosynthetic genes and transcriptional factors and potentially correlate them with metabolomics data to gain deeper insight into the regulatory mechanisms of flavonoids biosynthesis in relation to the genotype origin, growing seasons or experimental conditions. Changes in the content of flavonoids in *I. variegata* leaves influenced by light quality and quantity are most likely the result of complex changes in the expression of structural/biosynthetic and regulatory genes involved in their metabolism. High positive correlations among the analyzed flavonoids indicate possible coordinative regulation of biosynthetic enzymes of the flavanones, flavones, and flavonols routes at the transcription level. Results indicate that hydroxycinnamic acids and xanthones biosynthetic routes might also be under the control of the same regulatory machinery, although positive correlations between different polyphenolic classes (phenolic acids, xanthones and flavonoids) are less significant.

### 3.5. The Importance of a Proper Season and Genotype Sampling

Despite the difference in environmental factors present between sun-exposed and understory habitats of *I. variegata*, the effect of habitat demonstrated a significant impact only on the luteolin quantities. On the contrary, our results showed that significant divergence in quantities of the for majority of the analyzed compounds (naringenin, quercetin, rutin, luteolin, apigenin, and mangiferin glucoside) resulted from differences among genotypes. Concurrently, the absence of a significant interaction between treatment and genotype, for almost all observed compounds, indicates a lack of genetic variability of phenotypic plasticity to different light conditions. These results stress the significance of proper genotype sampling in studies that employ secondary metabolites as taxonomic markers. Since significant differences among genotypes mostly persist even in changed environmental or experimental conditions, this could influence the selection of individuals having favorable amounts of target secondary metabolites.

Data from this experiment also highlighted the importance of a sampling season since mean values of almost all target secondary metabolites were found to be the highest in spring and decreased at the end of the vegetative period. These data differ from several studies, where the highest concentrations of secondary metabolites were registered in summer [54,55,56]. This trend might be the result of a potential seasonal redistribution of secondary metabolites between leaves and rhizomes in the *Iris* species [57]. Therefore, further ecophysiological research on rhizomatous species dealing with metabolite accumulation might include metabolite profiling in both above- and below-ground plant organs.

Since *I. variegata* is highlighted as a valuable source of flavonoids and especially xanthones, selection of high-productive genotypes as well as proper harvesting period could be of major importance for pharmaceutical purposes.

## 4. Materials and Methods

### 4.1. Plant Material

**Figure 4:**
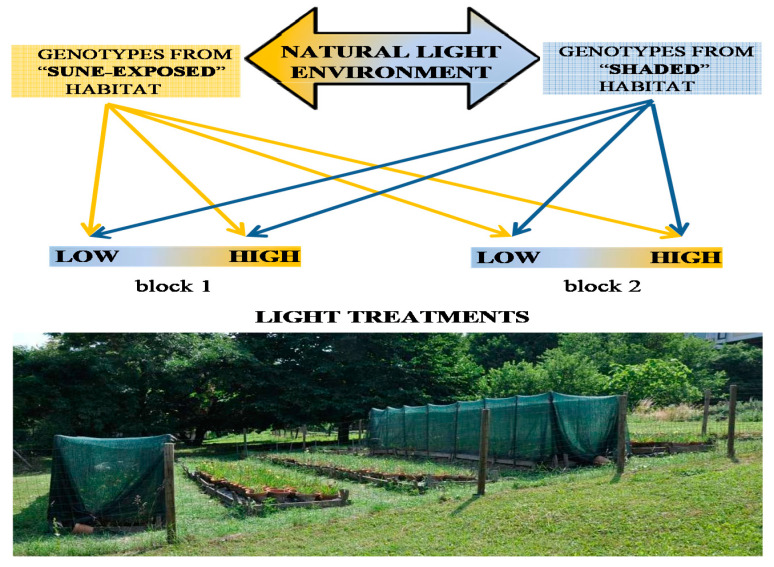
Experimental design at the experimental garden within the Institute for Biological Research “Siniša Stanković”, University of Belgrade, Serbia where a total of 68 genotypes of *I. variegata* were grown.

This research was performed on two groups of *Iris variegata* L. genotypes (68 in total) that originated in contrasting light conditions present in the dune system of the Deliblato Sands Special Nature Reserve (44°48′ N, 38°58′ E). The first group of genotypes (sun-exposed) occupied exposed areas on the dunes covered with annual and perennial herbs and low shrubs, while the second group of genotypes (shaded) was situated in the understories of diverse forest stands. The two habitats differed predominantly in terms of light intensity and light quality. Rhizome segments were taken to provide 4 clonal replicas of each of the 68 genotypes and were transplanted into clay pots (24 cm wide × 33 cm deep) in the experimental garden at the Institute for Biological Research “Siniša Stanković” National Institute of the Republic of Serbia, of the University of Belgrade (Serbia) (Figure 4). The experimental pots were filled with a common garden substrate (Premium Humus; Flora Company Ltd., Vranić, Serbia) and 1.0–2.0 mm quartz sand (Kvarc Company Ltd., Vlaško Polje, Serbia) and regularly watered. Four clonal replicas of each genotype were randomly assigned to one of the two experimental blocks, both including two light treatments: high, with photosynthetic active radiation (PAR) and R/FR ratio of 1625 μmol m^−2^s^−1^ and 1.07; and low, with (PAR) and R/FR ratio of 530 μmol m^−2^s^−1^ and 0.78 (Table S4). Experimental light treatments utilized in this study mimicked previously measured PAR values and R/FR ratio: dune areas with full sunlight (sun-exposed) of 1566 μmol m^−2^s^−1^ and 1.05 and woody areas with vegetative shadow (shaded) of 557 μmol m^−2^s^−1^ and 0.77 (Table S4). Contrast of light treatments was achieved by the use of green polyethylene shading net (Agrocentar Volodja Company Ltd., Pančevo, Serbia) that reduced light intensity and decreased R/FR ratio (Figure 4). During the experiment, the temperature was in average 1 °C higher in high light treatment compared to low light treatment.

### 4.2. Sample Preparation

Plants were allowed to acclimate during two successive growing years. Plant material (leaves from 34 genotypes originating from sun-exposed habitats and 34 genotypes from shaded habitats) was collected during spring, summer, and fall of the third year. Sampling was performed at the beginning of May, July, and September. The last fully developed leaf from each of the four clonal replicas was collected and dried in silica gel. Dry leaves were weighed, grounded in liquid nitrogen and extracted in 99.8% methanol (AppliChem, Cheshire, USA) (1:10 = *w*:*v*). Extraction was performed by vortexing for 1 min, and subsequently in an ultrasonic bath (RK100, Bandelin, Berlin, Germany) for 15 min. After centrifugation for 20 min at 10,000× *g*, the supernatants were filtered through 0.2 µm cellulose filters (Agilent Technologies, Santa Clara, CA, USA) and stored at 4 °C until use. Solvents for chromatographic analyses (acetonitrile and formic acid) were of HPLC or LC-MS grade and obtained from Fisher Scientific (Loughborough, UK). Methanol (HPLC grade) was purchased from AppliChem (Cheshire, CT, USA). Ultrapure water was generated by deionisation (Millipore, Billerica, MA, USA). Standards of phenolics (chlorogenic acid, caffeic acid, rutin, naringin, quercetin, luteolin, naringenin, apigenin, mangiferin) were purchased from Sigma-Aldrich (Steinheim, Germany).

### 4.3. UHPLC/(−)HESI-MS2 Metabolomic Profiling of Phenolics

The target metabolites were quantified in methanol extracts of *I. variegata* leaves in an single reaction monitoring (SRM) experiment using a triple-quadrupole mass spectrometer (TSQ Quantum access max, Thermo Fisher Scientific, Basel, Switzerland). Metabolites were chromatographically separated using a Dionex Ultimate 3000 UHPLC system (Thermo Fisher Scientific, Bremen, Germany) equipped with a Hypersil gold C18 column (50 × 2.1 mm) with 1.9 μm particle size (Thermo Fisher Scientific, USA). Analyses were performed at 30 °C. The mobile phase, i.e., 0.1% formic acid in water (A) and acetonitrile (B), was eluted according to the gradient previously published by Mišić et al. [58]. The flow rate of the mobile phase was set to 0.4 mL min^−1^. The injection volume was 10 μL.

A triple-quadrupole mass spectrometer with a heated electrospray ionization (HESI) source was set according to the following parameters: vaporizer temperature, 350 °C; spray voltage, 3500 V; sheet gas pressure, 28 AU; ion sweep gas pressure, 0 AU; auxiliary gas pressure, 4 AU; capillary temperature, 270 °C and skimmer offset, 0 V. Argon was used as the collision gas for collision-induced fragmentation and collision energy (cE) was set to 30 eV for all the target compounds. SRM analysis was performed using two diagnostic MS^2^ fragments for each compound.

External standards were used for quantification of the target metabolites: phenolic acids (caffeic acid, chlorogenic acid), flavonoids (naringenin, naringin, quercetin, rutin, luteolin, apigenin), and xanthones (mangiferin and mangiferin glucoside) in methanol extracts of *I. variegata* leaves. Preparation of stock-standard solutions was performed by dissolving 1 mg of a pure compound in 1 mL of methanol. Each of the stock solutions was adjusted to obtain the working standard solutions in concentration of 100 μg mL^−1^, that were subsequently diluted with methanol to obtain further calibration levels. The calculation of regression for each of the calibration curves showed excellent linearity, achieving correlation coefficients of *r* = 0.999 and *p* < 0.001. The total concentration of the analyzed compounds was obtained by calculating peak surfaces and was expressed in ng per 100 mg of dry weight of plant material (ng 100 mg^−1^ DW). Mangiferin glucoside was quantified relatively, using the calibration curve of mangiferin. Representative UHPLC/(−)HESI-MS2 total ion chromatograms are presented in Figure S1.

### 4.4. Statistical Analyses

The descriptive statistical analysis of the data obtained in this experiment was carried out using the means procedure of the SAS statistical package (PROC MEANS, SAS Institute, 2011) [59] for each of the analyzed characteristics. To investigate the effect of treatment, habitat, genotype and season on the phenotypic variation of the target compounds of *I. variegata*, analysis of the variance was applied. Prior to the analysis, data were Box-Cox transformed (STATISTICA, Statsoft, Inc. version 10). Since the experimental measurements of the same traits were repeated on each clonal individual over time (from the beginning until the end of the vegetation period), the obtained data were analyzed using a repeated measures analysis of variance (RM ANOVA, REPEATED option in SAS GLM procedure; SAS Institute, 2011) [59]. The RM ANOVA was computed for each trait in order to evaluate the following sources of phenotypic variation: treatment (phenotypic plasticity), block (microenvironmental variation), habitat (variation between different habitats), genotype (genetic variation nested within a habitat), treatment × habitat (plasticity variation between habitats) and treatment × genotype interactions (plasticity variation between genotypes). The analyzed individuals were referred to as subjects, the repeated observations on each individual as the within-subject or repeated factor, and the habitats and the experimental treatments as the between-subject factors. The season, season × treatment, season × habitat, season × genotype, season × treatment × habitat, and season × treatment × genotype interactions were considered as within-subject factors. A pattern of response of different compounds across the seasons was analyzed using the profile analysis (REPEATED/PROFILE option in the SAS GLM procedure; SAS Institute, 2011) [59]. The results of a profile analysis of the within-subject data showed whether there are significant changes in trait values in the following time intervals: between spring and summer, summer and fall, and spring and fall. 

The CORR procedure from the SAS package (CORR procedure; SAS Institute, 2011) [59], was used to estimate phenotypic correlations between trait pairs within both light treatments. Pearson’s coefficients of correlations were computed separately for each of the three seasons (spring, summer, and fall). Significant trait correlations were depicted by correlation diagrams in order to visualize the correlations patterns.

## 5. Conclusions

Phenolic profiles in *I. variegata* leaves were found to significantly differ between the two applied light treatments. Plants growing under a high light intensity and a higher red/far-red ratio contained significantly increased chlorogenic acid and mangiferin glucoside amounts, while these patterns were just the opposite for caffeic acid, luteolin and naringenin. Seasons during the vegetative period had a significant effect on these profiles with almost all secondary metabolites being most accumulated during spring, decreased in both summer, and fall, until the end of the vegetative period. Plants originating from sun-exposed and shaded habitats significantly differed in only one trait (luteolin), suggesting low genetic differentiation. The significant correlation among studied compounds were all positive, but the pattern and number varied between the two light treatments and seasons during the vegetative period, indicating the need for monitoring spatial as well as temporal environmental heterogeneity in estimating the species’ potential for evolutionary change.

## Figures and Tables

**Table 1:** Repeated measure analysis of variance (RM ANOVA) and PROFILE analysis for *Iris variegata* leaf secondary metabolites: phenolic acids (caffeic acid and chlorogenic acid), flavonoids (naringenin, naringin, quercetin, rutin, luteolin and apigenin) and xanthones (mangiferin and mangiferin glucoside) in genotypes from contrasting light habitats (sun-exposed and shaded), under two light treatments (low and high) and observed across seasons (spring, summer, fall) in one experimental year.

**Source of Variation**		**Flavonoids**						**Xanthones**			
		**Rutin**		**Luteolin**		**Apigenin**		**Mangiferin**		**Mangiferin gl.**	
**Between-Subject**	**df**	**MS**	**F**	**MS**	**F**	**MS**	**F**	**MS(×10^5^)**	**F**	**MS(×10^5^)**	**F**
Treatment (T)	1	0.03 × 10^−3^	0.00	10.58	6.47 *	1.22	2.54	24.79	0.03	90.09	11.95 ***
Block (B)	1	0.12	2.41	3.25	1.99	5.32	11.06 **	63.80	7.07	12.66	16.80 ****
Habitat (H)	1	0.03	0.68	10.08	6.17 *	0.02	0.05	70.68	0.08	82.83	1.10
Genotype (G(H))	45	0.11	2.30 *	7.74	4.73 ****	2.04	4.25 ****	20.42	2.26	17.09	2.27 ***
T × H	1	0.03 × 10^−4^	0.00	0.53	0.33	0.27	0.57	48.29	0.54	15.88	2.11
T × G(H)	15	0.06	1.23	1.84	1.13	0.44	0.93	93.01	1.03	67.17	0.89
Error	15	0.05	0.00	1.63		0.48		90.24		75.37	
Within-subject											
Season(S)	2	0.57	13.52 ****	233.88	156.61 ****	399.67	797.77 ****	40.90	66.67 ****	89.22	169.69 ****
S × T	2	1.15	27.21 ****	9.29	6.23	38.18	76.22 ****	31.93	5.20 **	27.27	5.19 **
S × B	2	0.11	2.69 ****	0.91	0.62	0.29	0.60	58.30	9.50 ***	47.34	9.00 ***
S × H	2	0.04	1.01 ****	0.27	0.19	0.21	0.42	20.78	3.39 *	51.24	0.97
S × G(H)	90	0.06	1.62 ****	2.71	1.82	1.07	2.15 ****	79.92	1.30	72.74	1.38 *
S × T × H	2	0.00	0.10 ****	2.45	1.65	0.19	0.40	78.38	1.28	17.24	3.28 *
S × T × G(H)	30	0.07	1.80 ****	1.36	0.91	0.51	1.01	77.87	1.27	57.99	1.10
Error	30	0.04		1.49		0.51		61.35		52.58	
Profile analysis											
spring–summer	1	1.61	65.44 ****	932.11	268.72 ****	1597.19	1646.24 ****	12.53	116.01 ****	13.30	116.20 ****
summer–fall	1	1.81	16.18 **	284.51	139.31 ****	358.20	548.22 ****	55.69	0.05	51.73	73.65 ****
spring–fall	1	0.01	0.05	186.66	54.12 ****	442.62	320.21 ****	12.01	87.87 ****	35.06	268.09 ****
**Source of Variation**		**Phenolic Acids**				**Flavonoids**					
		**Caffeic Acid**		**Chlorogenic Acid**		**Naringenin**		**Naringin**		**Quercetin**	
**Between-Subject**	**df**	**MS**	**F**	**MS**	**F**	**MS**	**F**	**MS**	**F**	**MS**	**F**
Treatment (T)	1	20.60	1235.80 ****	8.47	29.90 ****	1.14	37.97 ****	0.99	0.00	0.01	0.99
Block (B)	1	0.00	0.04	0.07	0.28	0.06	2.09	22.62	0.06	0.01	3.03
Habitat (H)	1	0.00	0.12	0.21	0.76	0.01	0.56	18.48	0.05	0.01	3.47
Genotype (G(H))	36	0.04	2.77	0.48	1.71	0.07	2.57 *	96.28	0.26	0.01	2.34 **
T × H	1	0.00	0.25	0.17	0.61	0.00	0.07	13.85	0.04	0.01	3.77
T × G(H)	1	0.14	8.75 *	0.11	0.40	0.02	0.68	22.48	0.06 *	0.00	0.92
Error	7	0.01		0.28		0.03		375.01		0.01	
Within-subject											
Season (S)	2	70.12	5448.54 ****	2.09	8.84 **	57.72	2268.62 ****	99.94	8.70 *	0.04	8.10 ***
S × T	2	4.88	379.81 ****	22.53	94.89 ****	0.96	37.81 ****	54.48	4.74 *	0.11	20.97 ****
S × B	2	0.09	7.40 **	0.17	0.75	0.14	5.71 **	571.67	49.77 *	0.05	9.57 ***
S × H	2	0.01	1.17	0.39	1.66	0.00	0.05	26.57	2.31	0.01	1.19
S × G(H)	72	0.05	4.62 **	0.15	0.64	0.06	2.66 **	129.86	11.31	0.01	1.04
S × T × H	2	0.00	0.32	0.02	0.11	0.00	0.07	297.75	25.92 *	0.01	1.07
S × T × G(H)	2	0.10	7.79 *	0.21	0.91	0.02	1.07	36.69	3.19 *	0.00	0.78
Error	14	0.01		0.23		0.02		11.48		0.01	
Profile analysis											
spring–summer	1	278.71	16,911.90 ****	2.69	5.40 *	220.40	3023.81 ****	199.89	8.70	0.09	9.22 **
summer–fall	1	90.46	3449.47 ****	1.55	3.45	21.32	4126.55 ****			0.15	12.22 ****
spring–fall	1	51.59	1494.70 ****	8.34	17.59 ****	104.60	1402.12 ****			0.01	0.65

* *p* < 0.05; ** *p* < 0.01; *** *p* < 0.001; **** *p* < 0.0001.

## Supplementary Materials

The following are available online at https://www.mdpi.com/article/10.3390/plants10081599/s1: Figure S1; UHPLC/(−)HESI-MS^2^ single reaction monitoring (SRM) chromatograms representing methanol extracts of *I. variegata* leaves (upper chromatogram) and pure standards (lower chromatogram). Corresponding MS^2^ spectra for each of the target compound are also presented: caffeic acid, chlorogenic acid, naringenin, naringin, quercetin, rutin, luteolin, apigenin, mangiferin and mangiferin glucoside. Table S1; Linear correlations (Pearson’s coefficients) between phenolic acids, flavonoids, and xanthones in *I. variegata* genotypes from contrasting light treatments (high—above the diagonal and low—bellow the diagonal) observed during the spring season. Table S2; Linear correlations (Pearson’s coefficients) between phenolic acids, flavonoids, and xanthones in *I. variegata* genotypes from contrasting light treatments (high—above the diagonal and low—bellow the diagonal) observed during summer season. Table S3; Linear correlations (Pearson’s coefficients) between phenolic acids, flavonoids, and xanthones in *I. variegata* genotypes from contrasting light treatments (high—above the diagonal and low—bellow the diagonal) observed during fall season. Table S4; Photosynthetic active radiation (PAR) and R/FR ratio in sun-exposed and shaded habitat (Deliblato Sands) and in contrasting light treatments (high and low light treatment) across three parts of vegetation period (spring, summer and fall).
