# Supplementary material for: Genetic and Environmental Factors Jointly Impact Leaf Phenolic Profiles of Iris variegata L
Source: Plants (Basel). 2021 Aug 4;10(8):1599. doi: 10.3390/plants10081599 (PMC8401273; doi:10.3390/plants10081599)
Supplement: Supplementary file 1 [file plants-10-01599-s001.zip › plants-1308049-supplementary.pdf]

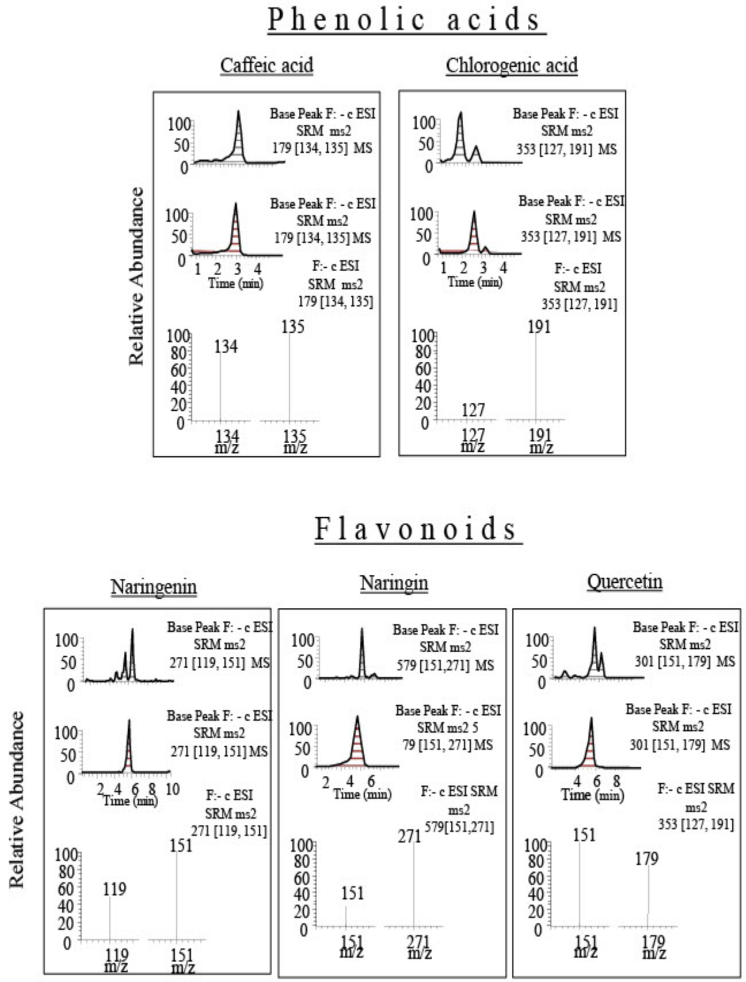

Phenolic acids
Caffeic acid
Chlorogenic acid
Base Peak F: - c ESI SRM ms2 179 [134, 135] MS
Base Peak F: - c ESI SRM ms2 353 [127, 191] MS
F:- c ESI SRM ms2 179 [134, 135]
F:- c ESI SRM ms2 353 [127, 191]
Time (min)
Relative Abundance
m/z
Flavonoids
Naringenin
Naringin
Quercetin
Base Peak F: - c ESI SRM ms2 271 [119, 151] MS
Base Peak F: - c ESI SRM ms2 579 [151,271] MS
Base Peak F: - c ESI SRM ms2 5 79 [151, 271] MS
Base Peak F: - c ESI SRM ms2 301 [151, 179] MS
F:- c ESI SRM ms2 271 [119, 151]
F:- c ESI SRM ms2 579[151,271]
F:- c ESI SRM ms2 353 [127, 191]

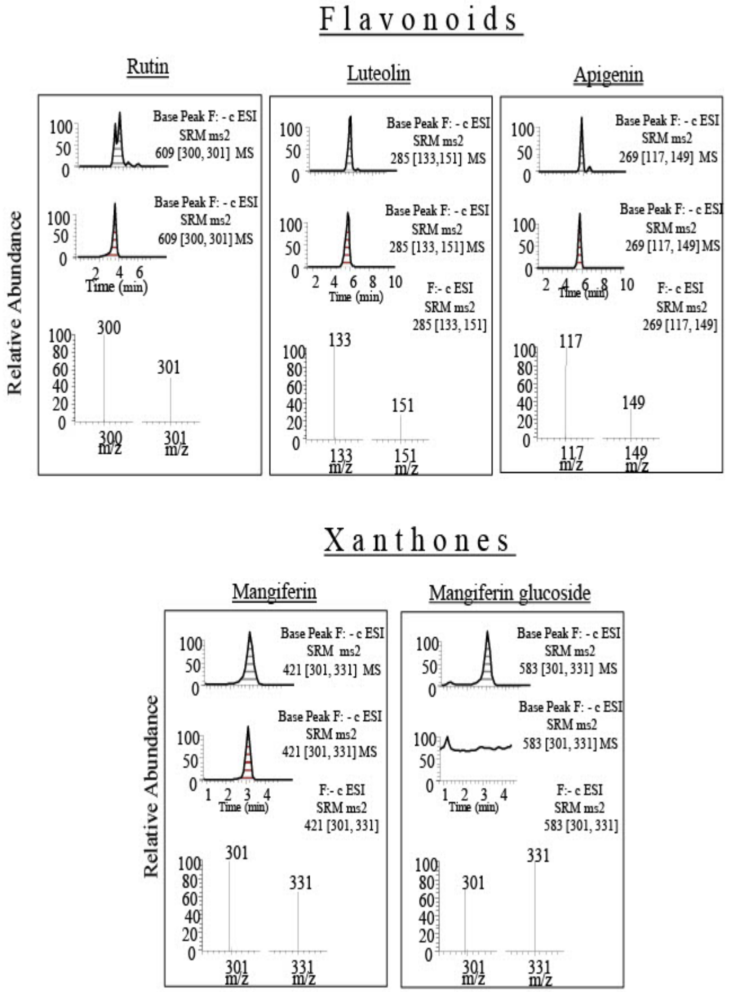


**Figure S1.** UHPLC/(−)HESI-MS$^2$ single reaction monitoring (SRM) chromatograms representing methanol extracts of *I. variegata* leaves (upper chromatogram) and pure standards (lower chromatogram). Corresponding MS$^2$ spectra for each of the target compound are also presented: caffeic acid, chlorogenic acid, naringenin, naringin, quercetin, rutin, luteolin, apigenin, mangiferin and mangiferin glucoside.

**Table S1.** Linear correlations (Pearson's coefficients) between phenolic acids, flavonoids, and xanthones in *I. variegata* genotypes from contrasting light treatments (High—above the di-agonal and Low—bellow the diagonal) observed during the spring season.

| | Phenolic acids | | Flavonoids | | | | | | Xanthones | |
|---|---|---|---|---|---|---|---|---|---|---|
| | Caffeic acid | Chlorogenic acid | Naringenin | Naringin | Quercetin | Rutin | Luteolin | Apigenin | Mangiferin | Mangiferin glucoside |
| Caffeic acid | | 0.41**** | 0.08 | 0.09 | 0.00 | 0.35**** | 0.30*** | 0.08 | 0.07 | -0.03 |
| Chlorogenic acid | 0.13 | | 0.03 | -0.05 | 0.21* | 0.27** | 0.22* | 0.01 | 0.22* | 0.13 |
| Naringenin | -0.07 | -0.05 | | -0.15 | -0.02 | 0.40**** | 0.15 | 0.41**** | 0.09 | -0.01 |
| Naringin | 0.14 | -0.11 | 0.58**** | | 0.52** | 0.21 | 0.09 | 0.14 | 0.22 | 0.03 |
| Quercetin | 0.12 | -0.08 | 0.36*** | 0.54*** | | 0.32*** | 0.14 | 0.01 | 0.37**** | 0.12 |
| Rutin | 0.11 | 0.16 | 0.41**** | 0.41**** | 0.19 | | 0.17 | 0.15 | 0.24** | 0.04 |
| Luteolin | -0.01 | -0.07 | 0.15 | -0.07 | 0.21* | 0.21* | | 0.53**** | 0.43**** | 0.17 |
| Apigenin | 0.14 | 0.05 | 0.36**** | 0.33* | 0.37*** | 0.28** | 0.62**** | | 0.17* | 0.04 |
| Mangiferin | -0.09 | -0.14 | 0.25** | 0.51**** | 0.37**** | 0.13 | 0.33*** | 0.27** | | 0.54**** |
| Mangiferin glucos | 0.18 | 0.07 | 0.08 | 0.33** | 0.30** | 0.23* | 0.24** | 0.32*** | 0.43**** | |

*P <0.05; ** P <0.01; *** P <0.001; **** P <0.0001

**Table S2.** Linear correlations (Pearson's coefficients) between phenolic acids, flavonoids, and xanthones in *I. variegata* genotypes from contrasting light treatments (High—above the diagonal and Low—bellow the diagonal) observed during summer season.

| | Phenolic acids | | Flavonoids | | | | | | Xanthones | |
|---|---|---|---|---|---|---|---|---|---|---|
| | Caffeic acid | Chlorogenic acid | Naringenin | Naringin | Quercetin | Rutin | Luteolin | Apigenin | Mangiferin | Mangiferin glucoside |
| Caffeic acid | | 0.29* | 0.00 | -0.06 | -0.03 | -0.04 | 0.17 | 0.14 | -0.01 | -0.01 |
| Chlorogenic acid | 0.15 | | -0.09 | -0.21 | 0.26* | 0.01 | 0.14 | 0.02 | 0.29** | 0.35*** |
| Naringenin | -0.02 | -0.07 | | 0.10 | 0.03 | -0.04 | 0.35** | 0.69**** | -0.03 | 0.00 |
| Naringin | 0.38 | 0.06 | 0.40 | | -0.18 | -0.22 | 0.02 | 0.24 | 0.36* | -0.05 |
| Quercetin | -0.11 | 0.11 | 0.01 | 0.29 | | 0.08 | 0.25* | 0.11 | 0.41**** | 0.42**** |
| Rutin | -0.06 | -0.05 | -0.08 | -0.19 | 0.01 | | 0.17 | -0.10 | 0.02 | -0.01 |
| Luteolin | 0.07 | 0.11 | 0.42**** | 0.29 | 0.27** | -0.09 | | 0.55**** | 0.32*** | 0.22* |
| Apigenin | 0.13 | -0.01 | 0.55**** | 0.36 | -0.03 | -0.13 | 0.61**** | | 0.21* | 0.15 |
| Mangiferin | -0.01 | 0.31** | -0.04 | 0.42* | 0.44**** | -0.07 | 0.37**** | 0.18* | | 0.60**** |
| Mangiferin glucoside | 0.03 | 0.26* | -0.07 | 0.48* | 0.33*** | -0.07 | 0.19* | 0.13 | 0.57**** | |

*P <0.05; ** P <0.01; *** P <0.001; **** P <0.0001

**Table S3.** Linear correlations (Pearson's coefficients) between phenolic acids, flavonoids, and xanthones in *I. variegata* genotypes from contrasting light treatments (High—above the diagonal and Low—bellow the diagonal) observed during fall season.

| | Phenolic acids | | Flavonoids | | | | | | Xanthones | |
|---|---|---|---|---|---|---|---|---|---|---|
| | Caffeic acid | Chlorogenic acid | Naringenin | Naringin | Quercetin | Rutin | Luteolin | Apigenin | Mangiferin | Mangiferin glucoside |
| Caffeic acid | | 0.21 | 0.02 | -0.56 | 0.05 | -0.04 | 0.01 | -0.02 | -0.01 | -0.05 |
| Chlorogenic acid | 0.11 | | 0.34$^{*}$ | 0.95 | 0.12 | -0.06 | 0.15 | 0.08 | 0.25$^{*}$ | 0.23 |
| Naringenin | 0.07 | -016 | | 0.84 | 0.25$^{*}$ | 0.01 | 0.56$^{****}$ | 0.57$^{****}$ | 0.28$^{**}$ | 0.01 |
| Naringin | -0.73 | 1 | -0.73 | | -0.11 | 0.05 | -0.50 | 0.11 | 0.31 | -0.78 |
| Quercetin | 0.03 | 0.09 | 0.26 | -0.72 | | -0.13 | 0.29$^{**}$ | 0.16 | 0.18 | 0.07 |
| Rutin | 0.09 | -0.07 | 0.72$^{****}$ | | 0.01 | | -0.18 | -0.13 | -0.01 | 0.10 |
| Luteolin | 0.28 | 0.05 | 0.65$^{****}$ | 0.26 | 0.18 | 0.12 | | 0.71$^{****}$ | 0.48$^{****}$ | 0.16 |
| Apigenin | -0.05 | -0.13 | 0.74$^{****}$ | 0.21 | 0.17 | 0.39$^{**}$ | 0.82$^{****}$ | | 0.42$^{****}$ | 0.19$^{*}$ |
| Mangiferin | 0.21 | 0.13 | 0.27$^{*}$ | -0.59 | 0.38$^{***}$ | 0.09 | 0.29$^{**}$ | 0.26$^{**}$ | | 0.54$^{****}$ |
| Mangiferin glucosi | 0.10 | 0.16 | 0.02 | 0.34 | 0.09 | -0.08 | 0.13 | 0.07 | 0.49$^{****}$ | |

*P <0.05; ** P <0.01; *** P <0.001; **** P <0.0001

**Table S4.** Photosynthetic active radiation (PAR) and R/FR ratio in Sun-exposed and Shaded Habitat (Deliblato Sands) and in contrasting light treatments (High and Low light treatment) across three parts of vegetation period (spring, summer and fall).

| | Deliblato Sands- natural habitat | | | | | |
|---|---|---|---|---|---|---|
| | Sun-exposed habitat | | | Shaded habitat | | |
| | Spring | Summer | Fall | Spring | Summer | Fall |
| Light intensity (µmol $m^{-2}$ $s^{-1}$) | 1797 | 1566 | 654 | 641 | 557 | 218 |
| R:FR | 1.06 | 1.05 | 1.08 | 0.96 | 0.77 | 0.63 |
| | Experimental garden | | | | | |
| | High light treatment | | | Low light treatment | | |
| | Spring | Summer | Fall | Spring | Summer | Fall |
| Light intensity (µmol $m^{-2}$ $s^{-1}$) | 1273 | 1625 | 1303 | 785 | 530 | 295 |
| R:FR | 1.02 | 1.07 | 1.05 | 0.80 | 0.78 | 0.71 |

*P <0.05; ** P <0.01; *** P <0.001; **** P <0.0001
